# Estrogen’s Tissue-Specific Regulation of the SLC26A6 Anion Transporter Reveal a Phenotype of Kidney Stone Disease in Estrogen-Deficient Females: A Systematic Review

**DOI:** 10.7759/cureus.45839

**Published:** 2023-09-24

**Authors:** Mawada Tarhuni, Monique N Fotso, Natalie A Gonzalez, Raghavendra R Sanivarapu, Usama Osman, Abishek Latha Kumar, Aishwarya Sadagopan, Anas Mahmoud, Maha Begg, Pousette Hamid

**Affiliations:** 1 Internal Medicine, California Institute of Behavioral Neurosciences & Psychology, Fairfield, USA; 2 Obstetrics and Gynecology, California Institute of Behavioral Neurosciences & Psychology, Fairfield, USA; 3 Pediatrics, California Institute of Behavioral Neurosciences & Psychology, Fairfield, USA; 4 Pulmonary and Critical Care Medicine, Texas Tech University Health Sciences Center, Odessa, USA; 5 Pulmonary and Critical Care Medicine, Nassau University Medical Center, East Meadow, USA; 6 Geriatrics, Michigan State University College of Human Medicine, East Lansing, USA; 7 Internal Medicine and Pediatrics, California Institute of Behavioral Neurosciences & Psychology, Fairfield, USA; 8 Internal Medicine, St. Joseph’s University Medical Center, Paterson, USA; 9 Neurology, California Institute of Behavioral Neurosciences & Psychology, Fairfield, USA

**Keywords:** oxalate homeostasis, calcium oxalate stone, kidney stone disease, pre and post-menopausal, estrogen, slc26a6

## Abstract

Kidney stone formation is an intricate process that involves a disruption in the interplay of the multiple organs and systems involved in regulating the concentration of specific ions in the body. Women who have gone through menopause are susceptible to kidney stone disease. This systematic review aims to investigate the potential influence of estrogen on kidney function and oxalate homeostasis, notably through the anion transporter SLC26A6 (also known as putative anion transporter 1 or PAT1) in females.

In accordance with the Preferred Reporting Items for Systematic Reviews and Meta-Analyses (PRISMA) 2020 checklist, a systematic search of online databases included Pubmed, ScienceDirect Journals, and Ingenta Connect Journals. Predetermined criteria to include and exclude papers, gathering articles published between 2012 and 2022, were determined. After a thorough analysis, eight articles (three cohorts, one case-control, one in vivo, one in vitro, and two cross-sectional studies) were identified for the final quality assessment review.

The eight selected and quality-assessed articles provided evidence of a directly proportional connection between estrogen and kidney function. A correlation between serum estrogen levels and the development of kidney stone disease was confirmed. Administration of β-estradiol was shown to effectively inhibit the function of the anion transporter PAT1 in a tissue-specific manner. In the case of the kidney, estrogen was observed to down-regulate PAT1, which led to a reduction in oxalate transporting activity and, consequently, a decrease in kidney stone formation. Consensus suggests that serum estrogen levels and optimal kidney functioning are interrelated. Furthermore, analysis of the quality-assessed articles and a comprehensive literature review revealed estrogen's tissue-specific regulation of the PAT1 anion transporter aids in maintaining kidney function and anion homeostasis. Additional research is needed to solidify estrogen's role in kidney stone disease to determine its therapeutic value in clinical practice.

## Introduction and background

Kidney stone disease

The development of kidney stones is a multifaceted, complex process. Etiology largely involves the multiorgan systems that uniformly regulate the homeostasis of relevant ions to dysfunction or be absent [1-5]. 

The major ions that contribute to the formation of calcium stones are calcium, phosphate, oxalate, and citrate. The most common type of kidney stone among the general population is calcium oxalate (CaOx), accounting for roughly 70% to 80% of cases [2]. Oxalate is unique amongst these ions in that the amount absorbed and excreted in urine is under little biological control [5]. In contrast, calcium and phosphate are subject to hormonal regulation in their absorption and excretion, whereas citrate excretion is primarily determined by renal transport and metabolism [3]. Gastrointestinal oxalate absorption is not known to be subject to regulation, and compared to the other ions, its absorption is less than 15% [3,4]. The oxalate absorbed is practically all excreted in the urine. A small portion (<10%) may be secreted back into the intestine, and if plasma oxalate levels rise above four μM, renal secretion of oxalate is thought to be stimulated [2,3]. Oxalate stone formation can be caused by the dysfunction of any part involving oxalate production and regulation [3-7].

Elevated levels of CaOx in urine, resulting from hyperoxaluria, have been identified as a notable risk factor for developing CaOx stones [3,6]. Hyperoxaluria is caused mainly by the oxalate metabolism defect. Risk factors linked to oxalate metabolic derangement include but are not limited to conditions such as obesity, hypertension, diabetes, and complications in organ systems like the gastrointestinal tract, liver, and kidney [6]. This Increased urinary excretion of oxalate is not only a significant risk factor for developing kidney stones but is also a biomarker for kidney stone development; the reduction of urinary oxalate is associated with a lower recurrent rate of CaOx kidney stones [8]. 

Oxalate production

Urinary oxalate is derived from two major sources; 80% to 90% comes from endogenous production in the liver, with the rest obtained from dietary oxalate [3,9-11]. AGT1 is a key enzyme in the liver in glyoxylate detoxification and is necessary to avoid oxalate formation from glyoxylate [3,9,12]. Typically, AGT1 converts oxalate precursors into glycine, an amino acid that the body can utilize safely or is excreted [3]. When AGT1 expression or function decreases, oxalate production is elevated in the liver, increasing renal CaOx crystal deposition. Primary hyperoxaluria type 1, a rare inherited metabolic disorder, results from a deficiency of AGT1 and leads to increased oxalate synthesis [4,5].

Estrogen preventing CaOx kidney stone formation via pathways AGT1

Studies have demonstrated that ERβ enhances hepatic AGT1 expression by directly binding to the 5' promoter of the AGT1 gene; in turn, leading to a decrease in the generation of endogenous oxalate, resulting in a reduction of urinary oxalate excretion and ultimately impeding the deposition of renal CaOx crystals [9].

Oxalate absorption, secretion, and excretion

Oxalate cannot be metabolized by the human body and is produced through various metabolic pathways. Its concentration in plasma depends on several factors, including dietary intake, intestinal absorption, metabolic production, and renal excretion [12]. The absorption of oxalate uniquely is not under hormonal regulation, and its absorption is less than 15% in the gastrointestinal tract compared to other ions [3,4]. Once absorbed, oxalate is then practically all excreted in the urine. A small portion (<10%) may be secreted back into the intestine, and if plasma oxalate levels rise above four μM, renal secretion of oxalate is thought to be stimulated [2,3]. The kidneys are accountable for eliminating 90% to 95% of endogenous oxalate [12]. The SLC26 gene family is responsible for producing anion exchangers that transport various anions, including oxalate [7,12]. Specifically, SLC26A6 (also known as putative anion transporter 1 or PAT1), A1, and A7 are the transporters that regulate the excretion and absorption of oxalate [12]. PAT1 is primarily found in the intestines and the apical membrane of the kidney. 

Role of PAT1 in intestinal absorption and secretion of oxalate 

The intestine plays a crucial role in restricting the absorption of oxalate. Oxalate is passively absorbed in the intestine through the paracellular pathway, and the net absorption of oxalate depends on the relative balance between the absorption of the paracellular pathway and the secretion of oxalate dependent on intestinal PAT1, mediating the secretion of oxalate in the intestine [10]. Studies have demonstrated that mice with hyperoxaluria, a condition characterized by excessive secretion of oxalate leading to increased urinary oxalate concentration, experienced significant improvement in their condition when adhering to an oxalate-free diet [13-15]. This suggests that the intestines are the primary source of urinary oxalates.

The transportation of oxalate is regulated by the concentration levels of chloride ions (Cl-) and bicarbonate ions (HCO3-) inside and outside of cells. This transfer is facilitated by PAT1, which mediates the exchange of these ions, allowing for oxalate transportation. Oxalate transport in the intestine can occur bidirectionally through transcellular and paracellular pathways. PAT1 is responsible for transcellular oxalate secretion, while absorption occurs through a paracellular channel. The distal colons of animals typically show basal net absorption of oxalate, in contrast to the basal net oxalate secretion in the small bowel and proximal colons. PAT1 in the mouse ileum facilitates apical oxalate secretion in exchange for Cl- [12]. Due to the intestine's strong connection with oxalate, certain factors such as intestinal microbes, type of oxalate, and fat absorption in the intestines have been found to interfere with oxalate absorption. A calcium-deficient diet can increase oxalate stone risk as calcium combines with oxalate to form CaOx, which is not easily eliminated. Adding calcium to the diet can reduce oxalate levels [3,4]. A high-fat diet and poor fat absorption can also increase free oxalate levels. Certain weight loss surgeries and digestive diseases can increase the risk of hyperoxaluria [3,4]. Taking fat-soluble vitamins can decrease oxalate excreted in the urine for those with poor fat absorption [3].* *

Studies have shown that a defect in the PAT1 can lead to hyperoxalemia, hyperoxaluria, and the formation of oxalate stones [13]. This occurs because the deficiency of PAT1-mediated oxalate secretion in the intestine leads to an increase in the net absorption of oxalate, which results in an increase in the concentration of oxalate in the plasma and a higher level of oxalate excreted in the urine. In contrast, the overexpression of PAT1 in the intestine can reduce the oxalate concentration in the urine and prevent kidney stone formation [13]. Therefore, regulating PAT1 expression could be a potential target for treating and preventing kidney stones.

Role of PAT1 in the renal excretion of oxalate

The kidney primarily eliminates most of the oxalate from the body [1-13]. While the glomeruli filter out much of the oxalate, the renal tubular secretion process further aids oxalate excretion [7]. In normal individuals, oxalate is freely filtered and not easily regulated, but the secretion of oxalate from renal tubules relies on the PAT1 protein [13]. Differences in PAT1 expression can impact oxalate secretion, affecting the regulation of oxalate in proximal tubules. PAT1 is responsible for transporting oxalate from cells to urine and vice versa. Studies show that the S1 and S2 segments of proximal tubules absorb oxalate while the S3 segment secretes it [13]. Abnormal expression of PAT1 can cause oxalate excretion disorders, leading to high oxalate levels in urine and the formation of oxalate stones [13]. Research has demonstrated that increased expression of PAT1 in the kidneys of mice can cause urinary oxalate concentration and stone formation rate to increase significantly. In vivo and in vitro experiments have also shown that increased PAT1 expression can lead to an increase in the secretion of oxalate in renal tubular epithelial cells, which can cause damage to the cells through oxidative stress [11]. Glycine has been found to reduce urinary oxalate excretion by down-regulating the expression of PAT1 in the kidney [16], which can help prevent the formation of oxalate stones [11,16]. Therefore, down-regulation of PAT1 is a potential strategy to prevent oxalate stones [17,18]. Recognizing that an imbalance in these processes can result in hyperoxaluria and hyperoxalemia allows for a clear understanding of why an increasing enteric oxalate secretion is a therapeutic target [17]. In contrast, excessive secretion can lead to a high urinary oxalate concentration [17,18].

## Review

Method and results

We searched electronic bibliographic databases (Pubmed, Omni Academic Search Tool: ScienceDirect Journals, Ingenta Connect Journals) (inception to 2012 to gain the most relevant and up-to-date findings and collect), relevant conference proceedings, tables of contents of journals, and review articles, all of which are detailed in Table 1.

**Table 1:** Electronic Databases Research Results _Omni Academic Search Tool was utilized to search and locate articles available through ScienceDirect and Ingenta Connect_

Search Results	ScienceDirect Journals: Advanced Search	ScienceDirect Journals: SLC26A6 Kidney Stone	Ingenta Connect Journals: Estrogen SLC26A6	Pubmed Advanced	Pubmed MESH
Initial	1,859	11	7	5,462	1,894
10YRS	602	5	6	699	266
English	598	5	6	694	263
Female	239	5	4	329	109
Free	106	4	4	1,518	409
Duplicate records	7				
Removed response	8,676				

**Figure 1:**
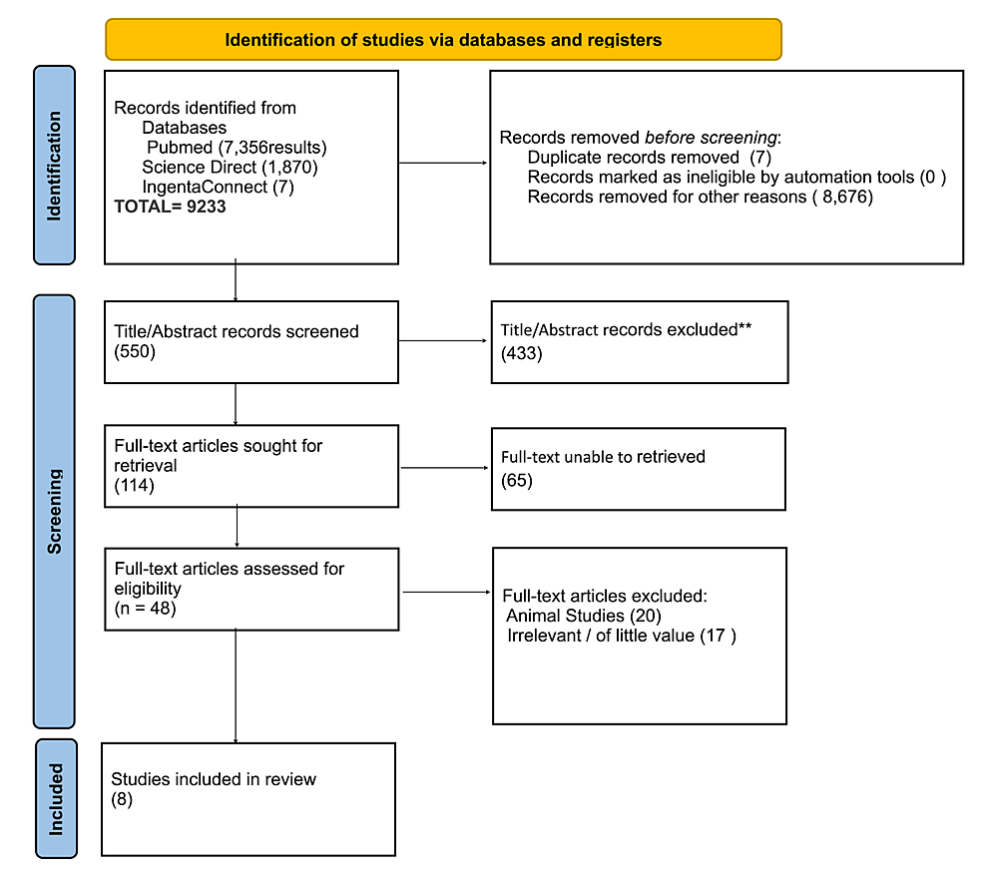
PRISMA Flow Chart of the Systematic Review

This was performed as per the 2020 Preferred Reporting of Systematic Reviews and Meta-analysis guidelines (PRISMA) and the results have been presented in Figure 1.

The population, intervention, control, and outcome (PICO) framework was utilized to conduct the searches, identifying the relevant patient or problems, the intervention under consideration, the comparison intervention, and the outcome measurements. The outcomes of these searches can be accessed in Table 2. Two authors independently screened the available literature to determine its relevance and ensure accurate data collection and interpretation. Literature was filtered to determine eligibility based on inclusion and exclusion criteria with consensus. Additionally, a third author was to resolve the difference of opinion.

**Table 2:** Search Techniques and Results _NCBI, National Center for Biotechnology Information; NLM, National Library of Medicine_ _Filters applied for all data: free full text, in the last ten years, English, female_

Database	Search Technique	Results
Pubmed	Keywords Building Block	SLC26A6 anion exchanger OR solute carrier family 26, member 6 protein OR Sulfate Transporters, anion-exchanger, anion transport proteins, oxalate Transporters, bicarbonate transporters Nephrolithiasis, Kidney stones, calcium oxalate stones, nephrolith, renal calculus, urinary calculus
Pubmed	Mesh Terms	(( "Sulfate Transporters/antagonists and inhibitors"[Mesh] OR "Sulfate Transporters/deficiency"[Mesh] OR "Sulfate Transporters/genetics"[Mesh] OR "Sulfate Transporters/physiology"[Mesh] )) AND ( "Sulfate Transporters/antagonists and inhibitors"[Majr:NoExp] OR "Sulfate Transporters/deficiency"[Majr:NoExp] OR "Sulfate Transporters/genetics"[Majr:NoExp] OR "Sulfate Transporters/physiology"[Majr:NoExp] ) (( "Kidney Calculi/etiology"[Majr] OR "Kidney Calculi/genetics"[Majr] )) OR ( "Kidney Calculi/etiology"[Majr:NoExp] OR "Kidney Calculi/genetics"[Majr:NoExp] ) (( "Nephrolithiasis/ethnology"[Mesh] OR "Nephrolithiasis/etiology"[Mesh] OR "Nephrolithiasis/genetics"[Mesh] )) OR ( "Nephrolithiasis/ethnology"[Majr:NoExp] OR "Nephrolithiasis/etiology"[Majr:NoExp] OR "Nephrolithiasis/genetics"[Majr:NoExp] )
NCBI at the U.S. NLM	Mesh Terms	SLC26A6 anion exchanger OR solute carrier family 26 OR member 6 protein OR Sulfate Transporters OR anion-exchanger OR anion transport proteins OR oxalate Transporters OR bicarbonate transporters OR Nephrolithiasis OR Kidney stones OR calcium oxalate stones OR nephrolith OR renal calculus OR urinary calculus AND (( "Sulfate Transporters/antagonists and inhibitors"[Mesh] OR "Sulfate Transporters/deficiency"[Mesh] OR "Sulfate Transporters/genetics"[Mesh] OR "Sulfate Transporters/physiology"[Mesh] )) AND ( "Sulfate Transporters/antagonists and inhibitors"[Majr:NoExp] OR "Sulfate Transporters/deficiency"[Majr:NoExp] OR "Sulfate Transporters/genetics"[Majr:NoExp] OR "Sulfate Transporters/physiology"[Majr:NoExp] ) AND (( "Kidney Calculi/etiology"[Majr] OR "Kidney Calculi/genetics"[Majr] )) OR ( "Kidney Calculi/etiology"[Majr:NoExp] OR "Kidney Calculi/genetics"[Majr:NoExp] ) AND (( "Nephrolithiasis/ethnology"[Mesh] OR "Nephrolithiasis/etiology"[Mesh] OR "Nephrolithiasis/genetics"[Mesh] )) OR ( "Nephrolithiasis/ethnology"[Majr:NoExp] OR "Nephrolithiasis/etiology"[Majr:NoExp] OR "Nephrolithiasis/genetics"[Majr:NoExp] ) Filters: Free full text, in the last 10 years, English, Female
NCBI at the U.S. NLM	Advanced Search Terms	(SLC26A6) AND (Renal) OR (Kidney) AND (Estrogen) Additional terms: SLC26A6 kidney stone, estrogen SLC26A6

Eligibility Criteria 

The selection process was conducted through the application of the PICO criteria framework, which involves the consideration of population, intervention, comparison, and outcome. The corresponding information is presented in Table 3.

**Table 3:** PICO Criteria for Developing a Research Question PICO: population, intervention, control, and outcome

PICO Elements	Keywords
​P: Population	Pre- and post-menopausal women
I: Intervention	Estrogen
C: Comparison	Pre- and post-menopausal women in context to developing kidney stone disease
O: Outcome	Estrogen prevents kidney stone disease by downregulating the anion transporter SLC26A6

Inclusion and Exclusion Criteria

Each selected article met the pre-established criteria, ensuring only relevant and informative content was included in the final selection. The considered criteria included the participants' gender, any interventions used (if applicable), and the resulting outcomes. To ensure consistency, we only included papers written and published in English, intervention studies that focused on age groups 30 and above, and papers that investigated the effects of estrogen on the SLC26A6 transporter and the development of kidney stone disease. Additionally, papers that were no more than a decade old included only female adults and contained either animal in vivo or in vitro studies were included. Furthermore, papers conducted in various regions across the globe have been incorporated. Lastly, the selection was restricted to full texts available for free or open-access papers. The exclusion criteria encompassed various aspects, such as excluding papers that solely focused on kidney stone disease non-specific to females, excluding papers that included male participants, papers that were classified as grey literature, papers that were translated from a language other than English, excluding publications that were not written in English, and excluding studies that were published over a decade ago. 

Quality Assessment

The evaluation of study designs was conducted by two reviewers who utilized recommended study-specific appraisal tools. Articles that exhibited high-risk bias were excluded, and only those with low-risk bias and good quality were selected for data extraction. Table 4 showcases the Newcastle-Ottawa for Cohort Studies, Cross-Sectional Studies, and Case-Control. Table 5 displays the articles evaluated using Cochrane Collaboration Quality Assessment Tools for Vitro/Experimental Studies. 

**Table 4:** Newcastle-Ottawa Quality Assessment Scale Cohort Studies/Cross-Sectional Studies and Case-Control Studies

Study	Selection	Comparability	Outcome	Risk of Bias
Zhao et al., 2013 [19]	****	*	**	Low risk of bias
Ahn et al., 2021 [20]	****	*	***	Low risk of bias
Kang et al., 2020 [21]	****	*	***	Good quality
Li et al., 2021 [22]	***	*	***	Good quality
Kattak et al., 2018 [23]	****	*	***	Low risk of bias
Faramand et al., 2021 [24]	****	*	***	Low risk of bias

**Table 5:** Cochrane Collaboration Quality Assessment Tools Used in Systematic Reviews of In Vitro/Experimental Studies

Criteria Cochrane Collaboration	Lee et al., 2022 [25]	Zhu et al., 2019 [9]
Sample		
The balanced baseline characteristics between intervention groups	Yes	Yes
Randomization		
Randomization of allocation sequence	Potentially yes	Potentially yes
Blinding		
Allocation sequence	Potentially yes	Potentially yes
Sample/participants	Potentially yes	Potentially yes
Investigators/assessors	Potentially yes	Potentially yes
Procedure		
Samples received a proper procedure	yes	Yes
Appropriate analysis	yes	Yes
Identical analysis between group	yes	Yes
Reporting outcomes		
Complete reported results	yes	Yes
Complete data	yes	Yes
No selection of reported results	yes	Yes
Missing data reported	Potentially yes	Potentially yes
Risk of bias	Low risk of bias	Low risk of bias

Data Extraction and Analysis

After a thorough evaluation by two independent reviewers, the final count of eligible articles was determined per the previously established criteria. From there, the content of the selected articles was meticulously structured in Table 6, and a diligent search for relevant information pertaining to the research topic was conducted. Then a categorization of the articles into two groups based on their content and thematic relevance to the research question was completed. 

**Table 6:** Outline of the Reviewed Article's Content and Results _ESRD, end-stage renal disease; SD, standard deviation; n, number; COS, calcium oxalate stone; ERβ, estrogen receptor beta; ERβKO, ERβ-knockout; PHTPP, 4-[2-phenyl-5,7-bis(trifluoromethyl)-3-pyrazolo[1,5-a]pyrimidinyl]phenol; NADPH, nicotinamide adenine dinucleotide phosphate; MHT, menopausal hormone therapy; RDL, reproductive lifespan duration; EEE, endogenous estrogen exposure; CKD, chronic kidney disease; ROS, reactive oxygen species_

Study	Study Design	Country	Results	Conclusion
Zhao et al., 2013 [19]	Case-control	China	The level of Serum E2 was found to be significantly lower in kidney stone patients compared to the control group (21.1 vs. 31.1 pg/ml). Post-hoc analysis revealed that this effect was mainly observed in patients with COS (p<0.001). Further analysis based on tertiles of E2 levels demonstrated a significantly higher frequency of COS in the group with the lowest E2 levels (p <0.001). A multiple logistic regression analysis identified the E2 level as a strong independent factor associated with the risk of COS. For every 1 SD increase, OR was found to be 0.951 with a 95% confidence interval of 0.919-0.985. The highest to lowest tertile OR was 0.214, with a 95%CI of 0.069-0.665. However, the groups had no significant difference in serum T levels.	Naturally, postmenopausal women with higher remaining estradiol levels appear less likely to suffer from kidney calcium oxalate stones. However, no correlation was found between serum T level and kidney stones. These findings support the hypothesis that higher postmenopausal endogenous estrogens may protect against kidney stones with aging.
Lee et al., 2022 [20]	In Vitro	Korea	The stimulation of β-estradiol decreased the transportation activities of oxalate or bicarbonate through SLC26A6. The activity of the transporter was reduced after the knockdown of SLC26A6, but cellular migration was increased. β-estradiol-mediated cellular migration was independent of SLC26A6 transporter activity. However, when the expression of SLC26A6 was increased, cellular migration was reduced even when β-estradiol was present.	These results indicate that β-estradiol treatment enhances cancer cell migration and dysregulates oxalate transport by inhibiting SLC26A6 activity, suggesting reduced oxalate transporting activity may involve oxalate homeostasis.
Zhu W et al., 2019 [9]	In Vivo	China	Mice without ERβ (ERβKO) and those treated with ERβ antagonist PHTPP exhibit higher renal CaOx crystal deposition levels, increased urinary oxalate excretion, and greater renal ROS production. In vivo mouse model, the use of the NADPH oxidase inhibitor, apocynin, to target ERβ-regulated NOX2 has been found to suppress renal CaOx crystal deposition effectively.	Together, results from multiple in vitro cell lines and in vivo mouse/rat models demonstrate that ERβ may protect against renal CaOx crystal deposition by inhibiting hepatic oxalate biosynthesis and oxidative stress-induced renal injury.
Ahn et al., 2021 [21]	Cohort	Korea	1,460,311 individuals participated and were categorized into four groups based on how long they used MHT: no MHT history, MHT use for less than 2 years, MHT use for 2 to 5 years, and MHT use for 5 or more years. Throughout the 9-year study, 4905 participants developed ESRD. Those who had previously used MHT had a 30% lower risk of developing ESRD. The findings from the subgroup analyses were comparable to those of the main study.	The findings in this study demonstrate the beneficial effects of MHT on the development of ESRD in postmenopausal women. Based on the results, our study may offer suggestions for further studies to investigate the therapeutic options for kidney disease.
Kang et al., 2020 [22]	Cross-sectional	Korea	In this analysis, the average age with standard deviation was 56.3±4.9 years, and the estimated glomerular filtration rate was 93.1±13.6 mL/min/1.73 m2. The mean RLD was 34.2±4.0 years. Among the 50,338 women studied, 765 (1.52%) were diagnosed with CKD. After conducting logistic regression analysis, it was found that the odds ratio for CKD was lower in groups with longer RLDs compared to the group with the shortest RLD. Over 9.7 years, postmenopausal women with normal kidney function were tracked in a longitudinal analysis. Out of 3155 participants, 221 (7.00%) developed incident CKD. Further analysis showed that the risk of CKD was lower in groups with longer RLD. This finding was significant even after adjustments for confounding factors.	The risk for CKD was lower in women with longer RLDs. The amount of endogenous estrogen exposure could be a determining factor for renal function in postmenopausal women.
Li et al., 2021 [23]	Cross-sectional	China	As women progress from pre-menopause to perimenopause and post-menopause, their renal function gradually declines. This decline is accompanied by an increase in the follicle-stimulating hormone (FSH) level in the bloodstream. In women who have gone through menopause, an increase in serum creatinine level was observed as the FSH quartile increased. This increase was accompanied by a decrease in the estimated glomerular filtration rate (eGFR) with a trend of p<0.001. Additionally, the prevalence of declined eGFR (<90 ml/min/1.73 m2) and chronic kidney disease (CKD; eGFR <60 ml/min/1.73 m2) also increased with a trend of p<0.001. The odds ratios (ORs) of declined eGFR and CKD increased in post-menopausal women, even after accounting for confounding factors. In the highest quartile of FSH, the odds ratios for declined eGFR (OR=2.19, 95% confidence interval [CI]: 1.63–2.92) and CKD (OR=10.09, 95% CI: 2.28–44.65) were about 2 and 10 times higher, respectively, compared to the lowest quartile of FSH (p<0.05). When post-menopausal women were divided by median age (61 years), the odds ratio for decreased eGFR was higher for each FSH quartile in the older group compared to the corresponding FSH quartile in the younger group.	A high circulating FSH level is an independent risk factor for renal dysfunction in women after menopause. Additionally, aging may aggravate the association of high FSH levels with reduced renal function in post-menopausal women.
Kattak et al., 2018 [24]	Cohort	United States	Women with both ovaries removed are at a greater risk of developing CKD as measured by eGFR (211 cases reported for women who underwent oophorectomy compared to 131 cases in the control group). The adjusted hazard ratio was 1.42, with a 95% confidence interval of 1.14 to 1.77, and the absolute risk increase was 6.6%. Women who had an oophorectomy at the age of 45 had a higher risk of developing CKD (with 110 events reported) compared to those who did not have the surgery (60 events reported). The adjusted hazard ratio is 1.59, with a 95% confidence interval of 1.15 to 2.19. The absolute risk increase is 7.5%.	Premenopausal women who undergo bilateral oophorectomy, mainly those 45 years old, are at higher risk of developing CKD, even after adjusting for multiple chronic conditions and other possible confounders present at the index date.
Faramand et al., 2021 [25]	Cohort	Iran	The overall rate of CKD occurrence over time was 50.1 cases per 1000 people per year, with a confidence interval of 47.7 to 52.6. Among women, the rates were 53.9 (CI 50.2-57.8) and 47.1 (CI 44.0-50.4) per 1000 person-years for those with less than or equal to 11 years and more than 11 years of EEE, respectively. When adjusting for age, BMI, smoking, hypertension, and diabetes, the model revealed that women with EEE < 11 had a hazard ratio (HR) of 2.66 (95% CI, 2.2, 3.2) for developing CKD compared to those with EEE ≥ 11 in the subgroup of women under 45 years old. In the subgroup of women aged ≥45 years, the HR was 1.22 (95% CI, 1.04, 1.4).	This study shows a higher HR of CKD incidence in women with low EEE levels later in life. Screening of these women for CKD may be recommended.

**Figure 2:**
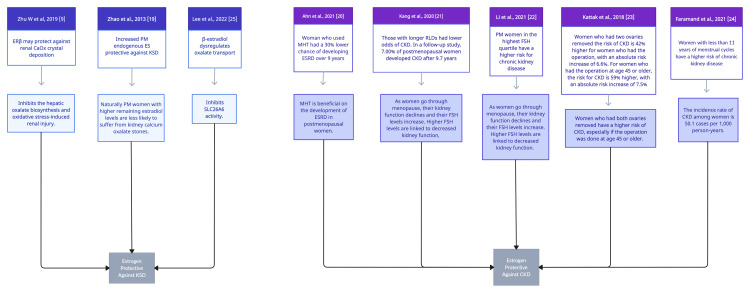
Categorical Thematic Synthesis of Quality-Appraised Articles _CKD, chronic kidney disease; RLD, reproductive lifespan duration; MHT, menopausal hormonal therapy; ESRD, end-stage renal disease; FSH, follicular stimulating hormonal; PM, post-menopausal; ERβ, estrogen receptor beta_

Figure 2 illustrates the categorization of the articles based on the synthesized findings to identify distinct themes. Further analysis and elaboration are provided in the forthcoming discussion section to address the research question effectively in various subheadings.

Discussion

The gathered quality articles from the databases all confirmed a robust, complex connection between estrogen and the SLC26A6 anion transporter does exist; although each article presented a different piece, all aligned to illustrate the relations. We classified the data collected into two distinct groups based on comparable outcomes. 

Categorization of the themes identified in quality-appraised articles

Estrogen and Kidney Stone Disease, Group One

Three of the eight studies indicated a direct correlation between estrogen levels and kidney stone disease development [9,19,25]. Low serum estradiol (E2) levels were significantly associated with a higher frequency of calcium oxalate stone (COS), and E2 was identified as a strong independent factor for the risk of COS [19]. Estrogen receptor beta (ERβ) was found to protect against renal CaOx crystal deposition by inhibiting hepatic oxalate biosynthesis and reducing oxidative stress-induced renal injury in vitro cell lines and in vivo mouse/rat models [9]. Mice lacking ERβ and those treated with ERβ antagonist PHTPP had increased renal CaOx crystal deposition, urinary oxalate excretion, and renal reactive oxygen species (ROS) production [9]. However, targeting ERβ-regulated NOX2 with the nicotinamide adenine dinucleotide phosphate (NADPH) oxidase inhibitor, apocynin suppressed renal CaOx crystal deposition in the in vivo mouse model [9]. Finally, β-estradiol was found to dysregulate oxalate or bicarbonate transporting activities by targeting the anion transporter SLC26A6, linking CaOx stone formation to the dysregulation caused by β-estradiol [25].

Estrogen and Kidney Function, Group Two

Analysis of the remaining five studies provided a consensus that indicates that estrogen levels and the optimal functioning of kidneys are interrelated [20-24]. Women who used menopausal hormonal therapy (MHT) had a 30% lower risk of developing end-stage renal disease (ESRD) [20]. Women with a longer reproductive lifespan (RLD) are at a decreased risk of developing chronic kidney disease (CKD) [21]. Out of 50,338 women, only 1.52% had CKD. In a follow-up study of postmenopausal women with normal kidney function, 7% developed CKD over 9.7 years. Cox analysis showed that longer RLD groups had a significantly lower risk for CKD development [21]. In postmenopausal women, higher follicle-stimulating hormone (FSH) levels were associated with increased serum creatinine and decreased estimated glomerular filtration rate (eGFR) [23]. The prevalence of declined eGFR and CKD also increased, even when accounting for cofounders; the likelihood of decreased eGFR and CKD rose when FSH levels were higher [22]. These associations were stronger in older females [22]. Women with both ovaries removed had a higher risk of CKD based on eGFR; the risk was exceptionally high for those with the procedure before age 45 [23]. Lastly, the incidence rate of CKD was 50.1 per 1000 person-years; women with less than 11 years of endogenous estrogen exposure had a higher incidence rate compared to those with 11 or more years. The study also found that the hazard ratio of incidence of CKD was higher in younger women with less endogenous estrogen exposure (EEE); age, BMI, smoking, hypertension, and diabetes were taken into consideration in adjusting the model [24]. All eight studies support estrogen's role and impact on kidney function and homeostasis. 

Two pathways of stone formation targeted by SLC26A6

Regulating Intestinal and Renal Oxalate Absorption and Secretion

The role of SLC26A6 in mediating epithelial oxalate secretion in the kidney is paradoxical to the role in mediating oxalate secretion in the intestine. The human body employs both renal and intestinal oxalate secretion mechanisms to effectively eliminate oxalate and subsequently decrease its concentration in the bloodstream, thereby reducing the overall amount present in the body. The absence of this gene has been shown to lead to the formation of Ca2+ oxalate stones due to hyperoxalemia and increased filtered load [12,13,26]. 

SLC26A6 plays a crucial role in preventing and treating kidney stones by regulating oxalate excretion [17]. When expressed in the intestine, SLC26A6 promotes oxalate excretion, reducing urinary oxalate levels and protecting against kidney stones [17,18]. On the other hand, when expressed in the kidney, SLC26A6 increases oxalate excretion, raising urinary oxalate levels and promoting kidney stones [26].

It is imperative to note that the SLC26A6 gene possesses the exclusive ability to remove oxalate from the intestine. While the SLC26A6-mediated oxalate secretion mechanism in the intestine is responsible for limiting the absorption of dietary oxalate and consequentially reducing urinary oxalate levels, it has been shown to have the opposite effect on the kidneys-increasing urinary oxalate levels [26]. 

Regulating Urinary Citrate

A6 also targets Ca+ oxalate stone formation pathophysiology caused by decreased urinary citrate. Maintaining sufficient citrate levels in urine is crucial in preventing calcium stone formation, regardless of calcium levels. One way to prevent stones is by using a transporter complex known as SLC26A6/NaDC-1 [26]. This complex serves a dual purpose in protecting against stone formation. Furthermore, SLC26A6 interacts with SLC13A2 or NaDC-1, another transporter in the proximal tubule SLC26A6 works with SLC13A2 to regulate the re-absorption of citrate in the proximal tubule [19,20]. This partnership is facilitated by the STAS domain of SLC26A6 and the f domain of NaDC-1 [26]. This interaction is crucial in inhibiting citrate uptake from urine, thereby regulating the rate and concentration of urinary citrate excretion [26]. 

To summarize, the study revealed that having too much SLC26A6 in the kidneys leads to higher oxalate excretion and urinary oxalate concentration, increasing the likelihood of developing kidney stones. Lowering the expression of SLC26A6 in the kidneys could be a promising way to prevent or treat urolithiasis [12,13]. To better understand the roles of intestinal and renal SLC26A6 in oxalate homeostasis, future studies should focus on tissue-specific deletion of SLC26A6.

Estrogen's paradoxical effect on SLC26A6

Studies have provided evidence that indicated estrogen has organ-specific varying effects on the anion transporter SLC26A6. 

Estrogen Upregulates PAT1 in the Female Reproductive Organs and Beyond

During the estrus cycle:* *The upregulation of PAT1 by estrogen was first demonstrated in mouse endometrium by He et al. suggesting that estrogen upregulates bicarbonate transportation/production proteins [27]. This provided new insights into understanding estrogen's role in regulating uterine bicarbonate transport [27]. The study found that the highest levels of the examined proteins were seen during “estrus.” The pH levels of the uterus were tested during both resting and active states and were found to be significantly higher during the resting state at estrus than during diestrus. The pH levels can be lowered through the use of blockers and inhibitors (blockers used: diphenylamine-2,2'-dicarboxylic acid; inhibitors used: 4,4′-diisothiocyanatostilbene-2,2′-disulfonic acid and acetazolamide). Estrogen was observed to increase certain expressions (CFTR, PAT1, CAR2, and CAR12), leading to an increase in the bicarbonate-dependent current and endometrial surface pH. These results were seen in both ovariectomized mice and endometrial epithelial cell cultures [27]. He et al. further elaborate, linking their findings; the uterus secretes bicarbonate and produces bicarbonate transport proteins differently throughout the estrous cycle in mice under normal conditions [27]. The highest protein expression level occurs during estrus, which aligns with the increased estrogen levels during this phase. Furthermore, shows that the bicarbonate transport proteins correspond with the pH measurements, with higher alkaline pH during estrus and lower pH during diestrus [27]. 

Bicarbonate Role In the Human Reproduction Pathway

In line with the known, well-established physiologic changes needed to regulate, optimize, and facilitate ovulation and reproduction. The fluid environment in the female reproductive tract is crucial for various reproductive processes, including sperm transport, fertilization, embryo development, and implantation. Disruption of this environment due to conditions like cystic fibrosis or hydrosalpinx can lead to infertility. Bicarbonate is essential for enhancing the mobility and fertilizing ability of sperm. During estrus, uterine bicarbonate secretion is increased, which is necessary for the fertilization process and overall fertility [27]. Besides its well-documented impact on the endometrium's growth, estrogen likely also regulates the uterine fluid environment by affecting its volume and bicarbonate content, as shown in this study's findings. Research shows estrogen is vital in stimulating uterine bicarbonate secretion during estrus, right before ovulation [28]. This is indicated by increased bicarbonate production, upregulation of transporter proteins, and results from pH measurements [28]. 

Bicarbonate Role in the Preimplantation of an Embryo

The female reproductive tract is heavily reliant on and influenced by HCO3 levels. Specifically, the oviduct and uterus have high HCO3− (up to 90 mM) levels, much higher than in other tissues. HCO3− entry into the preimplantation embryo activates soluble adenylate cyclase in the cytoplasm, leading to a series of events necessary for embryo cleavage. Insufficient secretion of HCO3- by the oviduct epithelium leads to the failure of embryo cleavage and blastocyst formation, which are crucial for embryo development [28].

Research showed that SLC26 A3 and A6 are expressed in embryos, and blocking their activity affects cleavage and HCO3-dependent events. Inhibiting/knocking down both exchangers has an additive effect similar to inhibiting CFTR [28]. Thus, the knocking down of SLC26A3, or SLC26A6, leads to reduced formation of cell embryos [28]. Both genes may play a role in embryo cleavage. It has been observed that the influx of HCO3− in preimplantation embryos serves as a cue for activating the expression of miR125b, which is crucial for the embryo's development through HCO3- dependent signaling [28].

To conclude, both He et al. and Lu et al. results suggest that the impact of estrogen extends beyond the growth of the endometrial lining and follicle development, as it also influences the movement and preparation of sperm and optimizes the uterine pre-ovulatory environment through the secretion of uterine bicarbonate [27,28]. Further, HCO3− signals activate miR125b expression in embryo development, which is crucial for growth. The alkaline pH's sensitivity to CFTR, SLC26A6, and CA inhibitors suggests that these proteins are functional and play a role in bicarbonate transport/production in the endometrial epithelium. These results indicate increased bicarbonate secretion during estrus and that estrogen may regulate the expression of proteins related to bicarbonate transport/production, forming a bicarbonate-rich fluid environment.

Small intestine*: *Endogenous estrogen increases the levels and functions of CFTR and SLC26A6 proteins responsible for transporting bicarbonate in the duodenum. This could explain why duodenal ulcers are less common in women than in men. Existing studies found that young women had higher expression levels of these proteins, which increased in the preovulatory phase of the menstrual cycle [29]. Ovariectomy decreased expression levels and bicarbonate secretion, which were reversed by estradiol supplementation [29]. Overall, findings from numerous studies support that estrogen upregulates the expression and function of these proteins, potentially explaining the gender disparity in duodenal ulcers.

Estrogen Downregulates PAT1 in Organ Systems Selectively Excluding the Female Reproductive Organs

Lung:* *Lee et al. 2022 reveal that increased estrogen levels can decrease the ability of A6 in lung cancer cells to transport oxalate or bicarbonate [25]. When the A6 protein is overexpressed, cellular migration is also reduced, even in the presence of estrogen [14]. Although the exact relationship between changed oxalate and calcium balance and the formation of stones is not entirely clear in this study, the results indicate that controlling oxalate movement through A6 may play a vital role in the connection between estrogen levels and kidney stone formation. Further, this suggests that the female hormones potentially can be a factor in the regulation of A6 activity involved in the formation of oxalate-related stones in humans. Additionally, hormone replacement therapy (HRT) may have therapeutic benefits in reducing oxalate-related stone formation- however, estrogen was shown to increase cancer cell movement in this study [25], so caution is necessary for cancer patients or those at risk as they may potentially be contraindicated. This does not come as a surprise, as HRT is considered a contraindication for patients with a history of breast cancer or other estrogen-based cancers like uterine cancer [30,31]. 

Kidney: Although estrogen's impact on A6 is still not concrete, significant evidence supports its reduction/nullification results in a reduction of CaOx stone formation, providing a blueprint to the complex physiologic pathways involved resulting in kidney stone disease. Research findings conclusively show that estrogen employs a dual mechanism to target A6 in the kidneys.

Estrogen Suppresses the Gene Expression of SLC26A6 in the Kidney

Previous differences in SLC26A6 expression in the proximal tubule between males and females have been expressed, with males showing dominance in this area. Studies done on male rats showed about 20% higher expression of rA6 mRNA in their kidneys compared to females. Further, this difference was confirmed at the protein level, with the rA6-related protein band being approximately 50% stronger in males. Immunostaining of kidney cryosections also showed that A6-Ab-related staining intensity was more substantial in males compared to females in both cortical and outer stripe tubules, indicating that the expression of rA6 is present primarily in male kidneys and further adding that it is influenced by androgen stimulation after puberty, and suggesting rA6 sex-dependent characteristics [32,33].

Estrogen Directly Inhibits/Reduces the Functioning A6 Transporter in the Kidney

ER signaling affects oxalate metabolism by inhibiting glycolate oxidase activity as well as SLC26A6 transporters [5]. Meanwhile, it also reduces a-enolase to reduce COM adhesion. Further studies suggest that estrogen treatment reduces the activity of highly dominant A6-expressed nephrons, leading to an imbalance of oxalate homeostasis and a decrease in transcellular oxalate secretion [25]. This could affect the secreted oxalate level and impact the regulation of oxalate secretion in the kidneys and other organs, such as the salivary glands and intestines, which highly express A6 [32].

Estrogen impact on the PAT1 co-transporter is still not concrete, and its metabolic effect has been described in parallel with other pathways where its reduction/nullification has been shown as the result of estrogen exposure, resulting in a reduction of CaOx stone formation. Metabolic changes in kidney stone disease. Estrogen receptor signaling affects oxalate metabolism by inhibiting glycolate oxidase activity and SLC26A6 transporters, which reduces a-enolase to decrease COM adhesion [25].

Functional investigations demonstrate the reduction of CaOx crystal‐binding capability of the estrogen‐treated cells consistent with the decreased levels of annexin A1 and α‐enolase (the known CaOx crystal‐binding receptors) on the cell surface [33]. High‐calcium and high‐oxalate challenges initially enhance the surface expression of annexin A1 and α‐enolase, respectively, which return to their basal levels by estrogen [33]. Additionally, estrogen reduces intracellular ATP levels and promotes cell migration and tissue healing [33]. Taken together, estrogen causes changes in the cellular proteome of renal tubular cells that lead to decreased surface expression of CaOx crystal receptors, reduced intracellular metabolism, and enhanced cell proliferation and tissue healing, all of which may contribute, at least in part, to stone prevention.

Liver:* *To date, no conclusive clinical research or trials have been conducted to ascertain estrogen's potential to downregulate PAT1 in the liver. However, we were able to provide a hypothesis rooted in the current knowledge and literature available, which suggests estrogen may down-regular A6 in the liver; further, we hypothesize that this connection may have therapeutic benefits in hepatocellular carcinoma (HCC). 

Estrogen Protective Against Hepatocellular Carcinoma

Research has shown that estrogen can help to prevent HCC, while androgen can increase the risk of liver and prostate cancer [34-39]; further, administering estrogen before treatment has been shown to protect the liver [35]. Estrogen can also inhibit ER-α-induced NF-κB signaling, slowing down the progression of HCC [36]. Moreover, estrogen can regulate the inflammation network in HCC by limiting proinflammatory cytokines and inhibiting downstream signaling pathways [36]. Although estrogen's anti-inflammatory properties have proven beneficial in protecting women from developing HCC, this is not the entirety of its complex benefits to the liver [36-38]. 

SLC26A6 Upregulated in Hepatocellular Carcinoma

Emerging research has concluded evidence that SLC26A6 is upregulated in HCC and is a better diagnostic marker than AFP [39]. Furthermore, high A6 expression is associated with shorter survival rates and poor outcomes in HCC patients, including neoplasm histological grade, stage, and TP53 mutation. SLC26A6 may play an oncogenic role in HCC progression and be a candidate clinical indicator. Steroid hormones may also play a role in regulating hepatic malignant transformation, adding that high estrogen levels in premenopausal women may protect against liver cancer and improve recovery after treatment [39]. Investigating ER expression and signaling is crucial due to the growing incidence of HCC worldwide; we further speculate that an increase in female lifespan may be a factor [40,41].

Compiling the recent study [39], which revealed that A6 exhibits potential as a "tumor marker" in HCC due to its upregulation in this disease and the extensive research that supports the role of estrogen as a preventive factor against HCC [34-38], is why we speculate that estrogen may downregulate A6, among its other protective functions against HCC. Additional research is necessary to verify the hypothesis mentioned. To our knowledge, we are the first to uncover this correlation, as currently, gaps in sources that explicitly support this, exist.

## Conclusions

The findings of this systematic review have validated our initial hypothesis that estrogen impacts the SLC26A6 (PAT1) ion transporter, specifically by down-regulating it, which possibly is the reason for the rise in the prevalence of kidney stone disease in estrogen females. Further, the outcome of eight studies that underwent quality appraisal and the thorough analysis of existing literature revealed that estrogen's regulation of the PAT1 transporter is organ tissue-specific. This was further supported by the alignment of the literature with the recognized physiologic changes caused by estrogen in the body. The findings have profound implications for all medical disciplines, given PAT1's direct involvement in multiple organ systems and their functional interdependence. To better understand the role of PAT1 in anion homeostasis and disease development, we advise future studies to focus on targeting tissue-specific deletion of PAT1 while simultaneously monitoring the influence of estrogen on this deletion. 
